# Supplementary material for: Global hypo-methylation in a proportion of glioblastoma enriched for an astrocytic signature is associated with increased invasion and altered immune landscape
Source: eLife. 2022 Nov 22;11:e77335. doi: 10.7554/eLife.77335 (PMC9681209; doi:10.7554/eLife.77335)
Supplement: Figure 2—source data 1. [file elife-77335-fig2-data1.zip › Figure_2_source_data_1/Figure_2A/homerResults/motif8.similar.html]

motif8

## Information for motif8

G
A
T
C
A
G
T
C
A
C
G
T
A
G
C
T
C
T
A
G
C
T
A
G
C
A
T
G
C
A
T
G
C
T
A
G
A
T
G
C
G
A
C
T
A
C
G
T
  
Reverse Opposite:  

T
G
C
A
C
G
T
A
A
T
C
G
G
A
T
C
G
T
A
C
G
T
A
C
A
G
T
C
A
G
T
C
T
C
G
A
C
G
T
A
T
C
A
G
C
T
A
G
  

|  |  |
| --- | --- |
| p-value: | 1e-25 |
| log p-value: | -5.955e+01 |
| Information Content per bp: | 1.759 |
| Number of Target Sequences with motif | 34.0 |
| Percentage of Target Sequences with motif | 1.46% |
| Number of Background Sequences with motif | 4.4 |
| Percentage of Background Sequences with motif | 0.12% |
| Average Position of motif in Targets | 90.9 +/- 53.3bp |
| Average Position of motif in Background | 111.2 +/- 40.3bp |
| Strand Bias (log2 ratio + to - strand density) | 0.2 |
| Multiplicity (# of sites on avg that occur together) | 1.03 |
| Motif File: | file (matrix) reverse opposite |

### Similar de novo motifs found

|  |  |  |  |  |  |  |  |
| --- | --- | --- | --- | --- | --- | --- | --- |
| Rank | Match Score | Redundant Motif | P-value | log P-value | % of Targets | % of Background | Motif file |
| 1 | 0.627 | G T A C G T A C A G T C C G A T A G T C A G T C A G T C A G T C C G T A T A G C C T A G A C T G | 1e-25 | -59.460697 | 1.16% | 0.08% | motif file (matrix) |
| 2 | 0.610 | G A T C G C A T A G C T C A G T A T C G A C G T T A G C C T A G A T C G T A G C | 1e-23 | -53.360541 | 1.08% | 0.08% | motif file (matrix) |
| 3 | 0.606 | C T A G A G T C A G T C C G T A C G T A A C T G | 1e-5 | -13.474670 | 12.23% | 9.29% | motif file (matrix) |
